# A Conceptual Model of Measurement Uncertainty in IoT Sensor Networks

**DOI:** 10.3390/s21051827

**Published:** 2021-03-05

**Authors:** Piotr Cofta, Kostas Karatzas, Cezary Orłowski

**Affiliations:** 1 Faculty of Telecommunications, Computer Science and Technology, UTP University of Science and Technology, 85-796 Bydgoszcz, Poland; 2 Environmental Informatics Research Group, School of Mechanical Engineering, Aristotle University, 54124 Thessaloniki, Greece; kkara@auth.gr; 3 Institute of Management and Finance, WSB University in Gdansk, 80-266 Gdansk, Poland; corlowski@wsb.gda.pl

**Keywords:** measurement uncertainty, IoT sensor networks, conceptual model

## Abstract

The growing popularity of inexpensive IoT (Internet of Things) sensor networks makes their uncertainty an important aspect of their adoption. The uncertainty determines their fitness for purpose, their perceived quality and the usefulness of information they provide. Nevertheless, neither the theory nor the industrial practice of uncertainty offer a coherent answer on how to address uncertainty of networks of this type and their components. The primary objective of this paper is to facilitate the discussion of what progress should be made regarding the theory and the practice of uncertainty of IoT sensor networks to satisfy current needs. This paper provides a structured overview of uncertainty, specifically focusing on IoT sensor networks. It positions IoT sensor networks as contrasted with professional measurement and control networks and presents their conceptual sociotechnical reference model. The reference model advises on the taxonomy of uncertainty proposed in this paper that demonstrates semantic differences between various views on uncertainty. This model also allows for identifying key challenges that should be addressed to improve the theory and practice of uncertainty in IoT sensor networks.

## 1. Introduction

Uncertainty is an important parameter of any measurement instrument. It determines its fitness for purpose, its perceived quality and the usefulness of information it provides. IoT sensor networks, the developing solution for environmental measurements, can be analyzed as complex instruments that deliver the reconstruction of the physical phenomenon. As such, it should be possible to determine the uncertainty of any such network as a whole. However, this is not the case. IoT sensor networks found themselves in a situation where established ways of dealing with uncertainty may not always be applicable, and where no unified theory of uncertainty in such networks can be found.

### 1.1. The Motivating Case

Let us consider citizen IoT networks that measure the level of air pollution such as the one described in [1]. These networks have been using some popular IoT protocols, mostly out of the COTS (commercial off-the-shelf) components. Such networks may consist of a few quality measuring nodes and an array of nodes that use various measuring protocols and are manned by volunteers at locations that are currently available. These networks may also employ some experimental algorithms before they are properly verified. While such networks may be inappropriate for industrial or professional settings, they are valid sources of information for several smart cities, and are the basis for important decisions. However, town halls increasingly face a question about the uncertainty associated with information delivered by such a network, and the answer to this question is far from being clear.

Such a network has several, often incompatible, uncertainties, originating from its various components. For example, there is an uncertainty introduced by sensors. There is also an uncertainty associated with the location of sensors in relation to the phenomenon. Data collection brings different forms of uncertainty, related to the operation of the network and data storage, as well as to the extent of possible malicious attacks. Next, there are uncertainties related to data processing, resulting from, e.g., the loss of precision, but also from the use of new or experimental methods. Other uncertainties are brought about by people involved in the design, development and operation of the network.

Were this an industrial control network or a professionally managed sensor network, both the definition of uncertainty (or uncertainties) in such a network, as well as the way of dealing with them would be well defined. This is because in such types of networks, the discussion about uncertainty has already reached a certain maturity and consensus, although restricted to a particular application area. For example, ref [2] discusses uncertainty of decision making for nuclear power plants while [3] deals with uncertainty applicable to the study of power systems. In the case of IoT sensor networks, the discussion is still open. Looking at the list of uncertainties provided above, the issue is not only that they differ by their origin or impact, but that they are often incompatible. There is no established approach and no shared taxonomy, nor is there a single understanding of what uncertainty is and how it can be measured or reasoned about. Furthermore, the relaxed standardization of both the equipment and procedures makes some established approaches to uncertainty inapplicable.

Referring back to the case of the IoT sensor networks [1], the authors determined, for example, that while the uncertainty introduced by sensors is acceptable, its key challenges lie in the long-term behavior of the sensors, specifically in the impact of the lack of reference measurements, calibration protocols and regular maintenance. Furthermore, increase in network-level uncertainty relates to longer term disconnection resulting from volunteers forgetting about the installation. Unfortunately, it was not apparent how to measure, consolidate and communicate uncertainty related to such varied sources as human actions, wear and tear as well as design faults.

This paper takes this situation as an opportunity. Every application area of sensing and measurement has gone through a similar situation and come out with its view on uncertainty, so that IoT sensor networks will eventually be no different. The primary objective of this paper is to facilitate the discussion of what progress should be made in relation to the theory and the practice of uncertainty in IoT sensor networks to satisfy current and future needs. 

### 1.2. Novelty and Benefits

Currently, there are several semantically incompatible research areas with their respective views on uncertainty, all partially applicable to IoT sensor networks. The key novelty of this paper lies in a systematic approach to the uncertainty of the IoT sensor networks, intentionally contrasting it with professional measurement networks. To this end, this paper contains the following novelty elements, all of which not only contribute to the body of knowledge, but are also expected to facilitate discussion that is beneficial for this research area:

- Identification of IoT sensor networks as a specific type of network, with their own challenges related to their uncertainty.
- Conceptual model of IoT sensor networks, as seen from the perspective of uncertainty while being specific to the conceptual architecture of the network.
- The taxonomy of uncertainty in IoT sensor networks that relates to the model while at the same time highlighting important differences in perceiving uncertainty in IoT sensor networks.
- List of potential research challenges, directly related to some current developments in the area of uncertainty and its treatment in IoT sensor networks.

This paper starts with discussion about the specificity of IoT sensor networks, already included in this section. It follows with the general discussion about the construct of uncertainty, as well as the overview of different approaches to uncertainty, with special focus on uncertainty in IoT networks. From there, it introduces the conceptual model of such networks, together with the matching taxonomy of uncertainty. The identification of key challenges is then followed by the case study that illustrates benefits of the approach described in this paper. Conclusions finalize the paper.

### 1.3. The Specificity of IoT Sensor Networks

The construct of uncertainty is applicable to any measurements, whether conducted as a direct observation or where complex instruments are involved. This section discusses the specificity of IoT sensor networks regarding uncertainty, proposing that their situation is specific enough to warrant a separate review of applicable approaches to uncertainty (see also [4] for a different analysis).

IoT sensor networks are a class of measurement instruments, specifically designed and operated in order to capture, process and reconstruct some physical phenomenon. Professional measurement networks (in contrast with the IoT sensor networks) are used throughout several application areas such as astronomy, physics or meteorology. For such networks, there are some established methodologies that deal with uncertainty, one of which is the information field theory [5].

**Figure 1:**
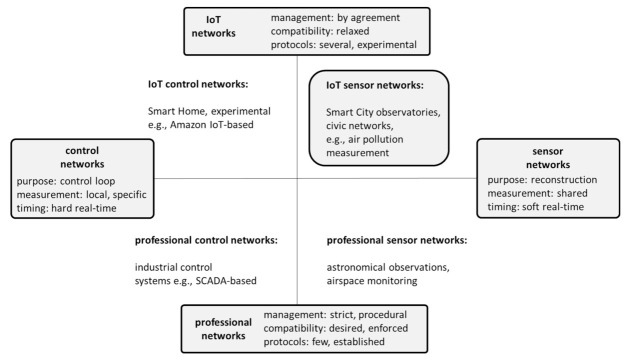
The specificity of IoT sensor networks.

This paper views IoT sensor networks as a specific combination of technology, management and application area that, in several aspects, differs from other networks. Intentionally contrasting IoT sensor networks, their differences can be analyzed along two axes, as shown in Figure 1.

- From the perspective of their purpose, they belong to sensor networks, i.e., networks whose purpose is to reconstruct certain physical phenomena. In contrast, control networks exist to implement the control loop that combines measurement with actions. Sensor networks tend to measure the physical phenomenon that is common to the networks (e.g., water quality, air pollution, etc.), not performing localized measurements characteristic of control networks (e.g., the measurement of the tension or movement). Sensor networks tend to benefit from a soft real time approach where the computation time is not a primary concern.
- From the combined perspective of technology and management, there is a distinction between moderated IoT networks and professionally managed networks. The approach of IoT networks to standards, quality of implementation or the observation of measurement protocols can be much more relaxed compared to professional networks. There can be several protocols in use, partially incompatible, while the operation can be performed by untrained volunteers. IoT projects tend to be more experimental and have lower budget. Partially, this situation is the outcome of the relative immaturity of the technology, and it may gradually improve.

IoT sensor networks found their applications mainly in projects related to the “smart” area: smart homes, smart cities, smart agriculture, etc., where the agility and low cost of solutions were considered the innovation enabler, while associated uncertainty was not a hindrance. However, as applications of IoT sensor networks grow in size and importance, uncertainty can become a forefront issue.

From the perspective of uncertainty, distinguishing features of IoT sensor networks are as follows:

- In the IoT networks, there may be more sources of uncertainty than in professional ones, specifically in places where the social perspective of uncertainty meets the technical one. Furthermore, the perception of uncertainty may vary among stakeholders.
- Variable provenance and quality of sensors, measurements and instruments render typical models of uncertainty not fully compatible with situations that exist in the IoT networks. Specifically, one cannot rely on a single model of a sensor, with its uncertainty tested under stringent conditions, but rather one has to deal with sensors of less prominent provenance.
- In the IoT networks, there may be less shared understanding of what the uncertainty is, how to express it, and how to minimize it, while professional networks benefit from the established knowledge accepted by all stakeholders.
- Measurements performed by IoT sensor networks are used to make decisions by bodies that are not skilled in asserting the quality of such measurements. Furthermore, those measurements can be used for various purposes, often ad hoc, in contrast with carefully thought-out, single-use measurements in professional networks.

All this may call for a separate effort to define what the uncertainty in IoT sensor networks is, and how to approach it.

## 2. Uncertainty—An Overview

Uncertainty is a pervasive concept, so that almost every discipline faces the challenge of incorporating it into its discourse. Consequently, it is almost impossible to discuss uncertainty “as such”, but always in the context of its usage, purpose or application area. The overview presented here is therefore divided into four parts. It first discusses the construct of uncertainty in general, but from the position that has been used throughout this paper. Next, it provides an overview of various approaches to make the discussion more systematic, usually specific to a given discipline or application area. The next section discusses various theories and metrics of uncertainty. Only then, this paper provides an overview of research in uncertainty that is specific to IoT networks.

### 2.1. The Construct of Uncertainty—An Overview

Uncertainty, in general, refers to epistemic situations where information, as received by the observer, is imperfect or may be unknown [6]. Consequently, only very unlikely situations where information is complete and perfect do not involve uncertainty. Uncertainty of a measurement of a physical phenomenon emerges in situations where the observer attempts to measure a certain aspect of the physical world. Such uncertainty can be considered a construct that is a result of an interplay between the subjectivity of the observer’s perception of the situation and the objectivity of the physical world. For example, subjectively, the observer may perceive the completeness or perfection of information about the measurement despite having neither of them. Whereas in reality, the complete information about any measurement is unattainable, being lower-bound by Heisenberg’s uncertainty principle. 

This interplay starts well before the actual measurement happens. The observers must be selective about what they want to measure from their physical environment. As the complexity of the physical environment is limitless, this process selects only some phenomena to be measured, and only some ways of measuring them. Thus, the “physical phenomenon” is identified primarily by the utility value as well as by its ability to be measured. For example, the phenomenon known as “PM10” (an air pollutant consisting of small particles with an aerodynamic diameter less than or equal to a nominal 10 micrometer) is constructed by clustering several pollutants together, and becomes of interest because its impact on human health is a running concern.

Only at this stage does the measurement network (including IoT sensor network) come into play. This paper views it as the intentionally constructed measurement instrument specifically designed to satisfy the observer’s (and the social system’s, represented by the observer,) desire regarding both the phenomenon and the way it is supposed to be measured. Thus, the function of the network is framed by the observer’s system to deliver the most reliable reconstruction of the defined phenomenon by using agreed means and methods, known collectively as protocols. 

The reliability of the reconstruction links back to the understanding of the phenomenon and the selection of protocols, and should be assessed only in this context. That is, every statement of the form “the value of PM10 is x”, should be fully qualified as “the value of the construct called PM10 as per given standard, measured according to a given protocol, is x”, where “x” is subject to some uncertainty.

Against this context, uncertainty emerges as a construct, attributable to the measurement instrument that represents the incompleteness or inaccuracy of information about the physical environment as compared to information that would have been required by the system. Some aspects of such uncertainty can indeed be attributed to the structure of the instrument, while others can be better attributed to the observer and the observer’s system.

The existence of uncertainty, and even the expectation of such existence, raises a doubt, be it about a particular measurement, the behavior of an instrument or the state of our knowledge in general. However, doubt that relates to uncertainty is not the uncertainty itself, and such doubt may be disproportional to the uncertainty. For example, even if very detailed information is available, thus suggesting low uncertainty, one may still doubt it as if not enough information has been presented.

Uncertainty differs also from error in that to speak of errors one has to observe a divergence between the actual and the expected, for example, between the value reported by the sensor and the value that is known to be true. In the absence of knowing the true value, the observer deals only with uncertainty, not with error. It should be noted that this situation can be subject to interpretation as to the attribution of the particular divergence to the error or to the uncertainty, depending on the frame assumed by the observer.

Another useful distinction is between uncertainty and risk. Risk is a calculative category that emerges where the probability distribution of a divergence is known and where the loss of utility can be associated with such distribution. In contrast, uncertainty emerges from epistemological situations where the distribution is not known, or where the utility is not attributable [7]. Therefore, measurements, contrasting with decisions, can be attributed only to uncertainty, even if the probability distribution function is known, because at this stage no utility can be defined. Decisions, however, deal with utility, so that ideally risk should be used, apart from situations when the distribution is not known [8].

One may also consider the second-order uncertainty, i.e., the uncertainty about the uncertainty itself. The first-order and the second-order uncertainty are usually expressed within bounds of the same theory. For example, in statistics, the second-order uncertainty is derived from the probability distribution function of possible probability distribution functions representing first-order uncertainties [9]. In a similar way, one may discuss the entropy of entropy, where entropy is taken as a measure of uncertainty [10].

### 2.2. Taxonomies and Approaches to Uncertainty

The brief overview below aims at demonstrating that the concern with uncertainty pervades all disciplines, not only natural sciences or those that deal with measurements. However, the approach to uncertainty that is visible throughout the literature is mostly segmented, with each discipline and application area striving to structure the discussion about uncertainty in a way that is most relevant to their needs. The very wide overview [11] indicates that each area must be selective or face the problem of excessive complexity of such taxonomy.

The attempt to unify various approaches to uncertainty is visible, e.g., in [3] that deals with a single application area (power systems) and purpose (uncertainty modelling). Two approaches to uncertainty are discussed: probabilistic and possibilistic (e.g., fuzzy sets), and a formal method to combine those two is presented. While no taxonomy of uncertainty is offered, it is suggested that those two approaches cover the majority of problems that the application area deals with.

Another application area (nuclear power plants) and another purpose (decision making) result in a different view on uncertainty [2], and on itself. Both the overview and the taxonomy (inspired by [12]) are presented that make a primary distinction between the objective and the subjective, and the model of transition from one form of the objective to another is proposed. There is also a good overview of approaches to uncertainty modelling.

Decision support for environmental problems has to deal with its own forms of uncertainty, mostly because this class of problems is difficult to reason about. An overview [13] reveals a number of approaches to dealing with uncertainty, such as expert assessment, model sensitivity analysis, the use of multiple models, etc. While the need to represent and reason about uncertainty is apparent, the paper does not provide a definitive answer on what method is most appropriate.

Health care has an apparent interest in uncertainty, as it increasingly uses evidence-based medicine. Failure to appreciate and comprehend uncertainty that comes with medical evidence may lead to incorrect diagnosis and treatment, potentially with fatal consequences. Unfortunately, there is no coherent approach to uncertainty. The three-dimensional taxonomy of uncertainty presented in [14] lists several other taxonomies that are present throughout medicine, and offers a new one that structures those approaches according to sources of uncertainty, issues (i.e., the domain or the purpose of the taxonomy) and locus (who or what is uncertain). It indicates that those three dimensions are interrelated, i.e., that, e.g., the identification of sources depends on issues. This paper comments that this taxonomy seems to be the only one that caters to different points of view (i.e., locus) on uncertainty, but it makes no efforts to integrate them into one view common to the whole discipline.

The concern related to uncertainty is also present in humanities, e.g., in the use of digitized sources [15]. The need for the new taxonomy is stimulated by the fact that humanities cannot directly reapply statistical methods used by natural sciences. The proposed taxonomy makes a primary distinction between intrinsic and extrinsic uncertainty, relating the former to the limitations of the subject (the “observer”) and the latter to uncertainties in data and their processing. This paper notes that this taxonomy is not very far off, conceptually, from the ones used throughout the natural sciences, only replacing views on uncertainty that are not applicable (most notably statistics) by those that are more suitable.

### 2.3. Uncertainty Theories and Metrics

Uncertainty is a pervasive concept, so it is not surprising that various theories of uncertainty developed independently, resulting in the mosaic of approaches. In the case of the IoT measurement networks, it is almost as if every component and every form of uncertainty has been used together with its own specific theory. 

Each theory tends to take its individual stance on what uncertainty is, and consequently it offers a metric for uncertainty that has different semantics, as well as rules for uncertainty propagation (fusion, computation) that are incompatible with other theories. The former is important when uncertainty has to be measured (e.g., at the sensors), the latter becomes important when uncertainty from various sources should be consolidated, e.g., during the reconstruction.

The brief overview of theories of uncertainty, presented below, does not claim to be a complete one. Its objective is mostly to demonstrate that differences between these theories are fundamental, as they define uncertainty with different semantics. For an interesting discussion see [2], for an in-depth overview see [16]. The overview attempts to address three questions: how does the theory interpret uncertainty (i.e., what is uncertainty according to the theory); what is the applicability of the theory for metrology and measurement networks; and what is the uncertainty propagation and fusion proposed by the theory.

#### 2.3.1. Probabilistic (Frequentist, Statistical) Approach

In the probabilistic (statistical, frequentist) approach, uncertainty is one of the parameters of the probability distribution function, usually its standard deviation. The measurement is seen as a process where the actual value is unknown, but its distribution can be estimated. Therefore, in order to estimate uncertainty, it is necessary to estimate the probability distribution function and then take its standard deviation or confidence interval as a metric for uncertainty. 

The probabilistic approach has become particularly beneficial in the area of sensors, where it can be used to determine their uncertainty in a systematic way. It is less applicable to situations where the probability distribution is not known, data is not available or where an appropriate environment to conduct a repetitive process of calibration cannot be easily created. Furthermore, information about uncertainty for values that are at tail ends of the distributions can be imprecise. On the conceptual level, the statistical approach is also sometimes criticized for its lack of self-doubt, as the value of uncertainty is taken as certain. There is ongoing research on overcoming its limitations, e.g., by combining it with the possibilistic approach [3].

This approach is very mature and its mathematical apparatus is widely accepted. Uncertainty propagation requires all components having their probability distribution function defined. In addition, it requires a formula that expresses the way components are combined into the final result. Under these conditions, statistical calculations, such as the combination of probability distribution (corrected for the effect of correlation) are to arrive at the final probability distribution function for which its uncertainty can be calculated.

#### 2.3.2. Bayesian (Empirical) Approach

The Bayesian approach is based on probability, yet it takes a very different view than the probabilistic one. In this approach, uncertainty is defined as a difference between the expected and the actual reading. That is, the process that produces predictable results has no uncertainty, even if those results are not identical. The Bayesian approach requires the construction of a model of the phenomenon that links observable readings to a set of parameters whose probability distributions are unknown and can be altered by evidence. Every new reading makes the model recalculate new distributions for all parameters to improve on its prediction capabilities.

The key advantage of this approach is that it always delivers the estimation of uncertainty, even if no reading has yet been made. Increasing the number of readings under identical conditions should result in a decrease in uncertainty, while changing conditions may be incorporated into the model. Unfortunately, this approach is sensitive to initial values of parameters, which may result in uncertainty that is unreasonably high or low.

Uncertainty propagation in the Bayesian approach can be a complex issue. It can use, e.g., the Gaussian process [17], with an alternative being the development of the complex model of the whole system and the direct application of Bayesian reasoning to it. It should be noted that the Gaussian process requires any subset of components to have a multivariate normal distribution; the requirement sets limits to this approach.

The Bayesian approach gradually found its place in metrology [18] as a substitute for the purely statistical approach, specifically in situations where repeatable measurement under identical conditions may not be achievable, but where initial distributions of parameters can be estimated. It should be noted that Bayesian statistics can also be used together with the subjective approach (discussed later) and with evidence theory, e.g., see [19].

#### 2.3.3. Approach Based on Evidence Theory 

Dempster–Shafer evidence theory [20] brings the general framework for reasoning under uncertainty, thus being close to imprecise probability theories. The theory allows one to combine evidence (e.g., past measurements) to arrive at the proposition that best takes into account such evidence, e.g., regarding the actual value. It is a primary framework to deal with epistemic uncertainty that has developed over the years.

The theory equates uncertainty with the lack of provability. That is, there are several propositions, and each piece of evidence can support a proposition with its weight. However, there is a differential weight of evidence that points to no particular proposition, and such weight is the level of uncertainty in the outcome of the reasoning. Thus, the proposition that cannot be proven (e.g., because there is as much evidence “for” as there is “against”) bears high uncertainty, while the proposition that is clearly supported by all evidence is certain. 

The connection between uncertainty and the evidence theory is still debated. For example, [21] demonstrates how the Dubois entropy can be analyzed using evidence theory in expressing uncertainty, but only under some conditions.

Dempster–Shafer theory application has been found to be a widely accepted evidence-based reasoning framework, specifically that its rules of combination could be effectively used for uncertainty propagation. There are some examples of this framework delivering counter-intuitive results (e.g., [22]), mostly because it concentrates on provability rather than on truth, i.e., it promotes propositions that can be proven (i.e., of low uncertainty) [23].

#### 2.3.4. Possibilistic Approach (Fuzzy Logic)

The possibilistic approach attempts to ascertain the value of uncertainty by calculating the possibility and the necessity of various outcomes, delivering a degree of belief in a given proposition. The formalization linguistic variables used to describe the situation can be done, e.g., through fuzzy logic [24]. Fuzzy logic is a version of many-valued logic that is used in situations where either input or output is imprecise, e.g., where it is based on linguistic variables. Any input value can belong to several fuzzy sets at the same time, without creating contradictions. Truth about belonging can be any real number, usually between 0 (false) and 1 (complete truth), while uncertainty is a distance between such truth and the complete truth.

Fuzzy logic is useful in situations where input can be expressed only in a vague way. For example, it can be used in metrology to aid the expression of uncertainty [25]. It is criticized for the introduction of subjectivity in the form of subjective language, rules for conversion into numerical values (i.e., fuzzification) as well as rules that govern fusion and reasoning.

Uncertainty propagation under fuzzy logic tends to follow the fuzzy reasoning process that can use fuzzy logic statements expressed, e.g., in a form of a set of rules [26]. The uncertainty associated with the system can be estimated from information available just prior to defuzzification. It is also possible to have the defuzzification process that takes uncertainty into account [27].

#### 2.3.5. Interval Arithmetic Approach

Interval arithmetic is a technique that replaces error distribution function with ranges (intervals), so that all values within the range are equally valid. Consequently, it interprets uncertainty in a binary way, disposing of any degree of uncertainty. Every result whose value falls into the interval is equally certain, every one outside of this interval is equally uncertain. Interval arithmetic can be used alone, or it can be combined, e.g., with fuzzy logic or linear algebra [28].

Interval arithmetic originated from statistical methods and bears close resemblance to confidence intervals. However, the computational simplicity of interval arithmetic allows for its use in situations where extensive computations are not attainable, such as tracking component tolerances or in optimization algorithms.

One should also note the interesting combination of Dempster–Shafer approach, Shannon entropy and the interval arithmetic [29], where uncertainty intervals are calculated using the information-theoretic approach but are combined according to the evidence theory. 

#### 2.3.6. Information-Theoretic Approach

The information-theoretic approach (e.g., [6]) links uncertainty to epistemic situations where information is imperfect or may be unknown. Only situations where information is complete and perfect bear no uncertainty. The information-theoretic approach is particularly useful when the situation can be expressed in terms of communication, e.g., when the measurement instrument such as a network can be thought of as a kind of communication channel.

Entropy is used here as a metric. Classical Shannon’s entropy as well as several other variants of entropy can be used. The close conceptual relationship between these two constructs is known [30]. As the complete information may be limitless (e.g., information about a physical phenomenon), uncertainty can also be quantified using the amount of information that can be practically obtained.

Uncertainty propagation is handled by operations on entropy, e.g., through the computation of the entropy of a convolution of functions, each function representing a single component. More advanced approaches lead, e.g., to entropy algebras [31], demonstrated for von Neumann entropy.

#### 2.3.7. Subjective Approach

The subjective approach to uncertainty takes a very different route, as it takes doubt expressed by the observer as a measure of uncertainty. Such a metric is both general and subjective, as it depends on the generalized impression of the observer. In order to achieve certain objectivity, the process of consolidation of opinions is an essential part of this approach. See [32] for an in-depth analysis of this approach.

This approach assumes that there is a population of observers who can form their opinions independently. Even if observers are non-experts, the “street-level epistemology” [33] may be sufficient. Despite its shortcomings, the subjective approach is valuable when other sources of information are not readily available; for example, when sensors cannot undergo calibration, the network growth is not controllable or where measurements use different protocols. 

The subjective approach can use one of the established frameworks for uncertainty propagation, e.g., fuzzy logic or evidence theory. It is also used together with socially inspired frameworks that introduce concepts of opinion and reputation as subjective and objective metrics for certainty [34]. Algorithms such as exponentially weighted moving average (EWMA) can be used to consolidate individual opinions into reputation [35].

### 2.4. Uncertainty in IoT Networks—An Overview

The problem of uncertainty in IoT networks (whether sensor networks or not) has become important over recent years, specifically when combined with the development of low-power wireless sensor networks. This brief overview indicates some of the key research directions. Due to the extensive nature of the problem, this section is not intended as a complete overview, but it focuses on various aspects of uncertainty that can be identified in modern sensor networks. 

The development of wireless sensor networks made uncertainty considerations quite important, due to the visible impact of uncertainty associated with the network. The design aspects of uncertainty in wireless sensor networks are addressed in [36], developing the uncertainty-aware deployment algorithm based on the Bayesian probability theory. The objective is to assure area coverage while taking into account uncertainties associated with the characteristics of the environment as well as properties of sensors. 

In [37], a soft-computing approach is proposed to determine the uncertainty of wireless sensor networks for the purpose of managing their quality of service. The proposed algorithm thus reaches beyond the technical or regulatory perspectives on uncertainty by incorporating some non-functional requirements. It uses rough set theories to reason about uncertainty in data sets created by such networks. 

The coexistence of various measuring instruments of varied quality is analyzed, e.g., in [38]. Uncertainty in wireless sensor networks used for traffic safety is analyzed, providing a detailed study regarding uncertainty associated with various types of measurements. The list of various sources of uncertainty is provided, as well as mathematical models of the uncertainty of given measuring instruments.

There are models of uncertainty specifically developed for the purpose of sensor networks. For example, [39] has developed a model of uncertainty for wireless sensors that takes into account, among others, the impact of environmental factors such as temperature and battery status, as well as possible failures.

Another approach to uncertainty is through simulation that allows experiments that estimate uncertainty associated with the network as well as its sensors. [40] developed a modular open-source software simulation tool to facilitate experiments in analyzing uncertainty for the purpose of sensor network design. Various conditions such as a sensor failure or noise can be simulated.

The IoT network is not only about sensors and transmission, but mostly about data. Uncertainty in sensor networks from the perspective of databases and their processing has been studied in [41], indicating that it is necessary to associate uncertainty with data collected, and to incorporate the handling of uncertainty into data processing.

In [42], the focus has been on uncertainty of data, from the perspective of model-driven reconstruction, to minimize the traffic in networks. New data are sent from the network only if it is recognized that the model used for data reconstruction and control approaches the undesirably high level of uncertainty. Furthermore, only nodes that may decrease such uncertainty to the acceptable level are allowed to send new data.

The security in IoT networks is a large research area, specifically focusing on the issue of identifying malicious nodes. A useful overview has been provided in [43] that discusses several forms of attack and proposes countermeasures. Another overview [44] focuses on IoT applications in smart cities, concentrating on appropriate architectural choices. An overview specific to a wireless sensor network, together with proposed countermeasures, has been provided in [45].

Several methods to detect malicious nodes are proposed and tested, with the intention to decrease the uncertainty of the network by eliminating or containing the impact of such nodes. Specifically, there are a large number of solutions that are based on “soft” assessment, i.e., on attributing the node with a level of trust that relates to the perception of whether it is malicious or not. An overview of such methods for wireless networks has been provided in [46], while an overview of techniques related to IoT networks can be found in [47]. It should be noted that in general these methods make no specific distinction between permanently faulty nodes and malicious ones.

## 3. The Conceptual Model of Uncertainty in IoT Sensor Network

This paper treats measurement networks (including IoT sensor networks) as purpose-built measurement instruments. The model presented in this paper provides a conceptual decomposition of the network into its components from the perspective of sources of uncertainty. While it was developed with the IoT sensor networks in mind, it is also applicable to professional measurement networks. The model also provides a link between the network components and various forms of uncertainty that are themselves structured into a taxonomy. Finally, it allows for the identification of research challenges associated with those components. 

### 3.1. The Conceptual Model of IoT Sensor Networks

**Figure 2:**
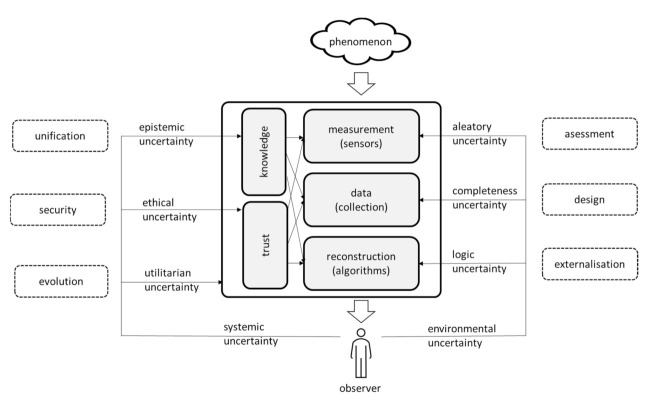
The sensor network as a measurement instrument.

Figure 2 presents the conceptual reference model of the measurement network, used specifically to structure the discussion about uncertainty (the central part of Figure 2) as perceived by the observer. The network is treated as the measurement instrument by which the observer (at the bottom) perceives a physical phenomenon (at the top). The objective of this instrument is to deliver the reconstruction of the phenomenon in a way that is true to the phenomenon and is comprehensible to the observer. 

For the purpose of the reconstruction, the instrument operates a three-stage process: measuring the phenomenon through sensors, collecting and processing data and finally reconstructing the phenomenon to be presented to the observer. The whole process is governed by the body of knowledge that is relevant to the network, by the way it operates and the guided observer’s decisions regarding the selection of the phenomenon and relevant protocols.

It should be noted that the model is intentionally conceptual, at a relatively high level of abstraction. It aims at uncovering the variety of approaches to uncertainty. It must be appreciated that detailed analysis of network components (such as network nodes, communication means, data storage, processing units, etc.) may reveal a more complex array of places where uncertainty has to be dealt with. However, it is unlikely that such an analysis will reveal more distinct approaches to uncertainty than this one does. Instead, this model concentrates on the observer’s perception of the network, as it guides the observer’s view on its uncertainty.

### 3.2. The Taxonomy of Uncertainty for IoT Sensor Networks

When uncertainty is considered, it can be seen from the literature review that there are various forms of uncertainty that the observer may consider within the network. Those uncertainties form the taxonomy presented below, but they also relate to specific components of the model, as shown in Figure 2. The problem of uncertainty is so pervasive that all aspects of uncertainty may be somehow visible in all components, but it is the intentional simplification of the model to attribute every aspect of uncertainty to a specific component where the given aspect is most visible.

There are several possible taxonomies of uncertainty. For an extensive overview, see [11]. This taxonomy has been developed specifically to address the observer’s view on uncertainty of the network. In contrast with [12] and its elaboration [2], it is not concerned with the decision-making process. This taxonomy takes the information-theoretic approach to uncertainty [6] and is inspired by the theory of social systems [48], specifically by the distinction between the system (the observer) and its environment (the physical world). 

This taxonomy makes a primary distinction, that can be found in several taxonomies (e.g., [2]), between the objective (i.e., environmental) and the subjective (i.e., systemic) ones. The approach to develop such a taxonomy is based on the concept of a social system (i.e., the observer) and its environment (i.e., the network and the physical world), combined with the understanding of the triple nature of such an environment: physical, technical and social, and a triple perspective on the social system: internal, external and reconstruction-oriented. As a result, it differs, e.g., in regarding the epistemic uncertainty as the subjective one, as this uncertainty is considered to be internal to the observer’s system. 

The taxonomy is shown in Figure 2 as a series of lines that lead from the observer to various elements of the model, indicating places within the model where particular aspects of uncertainty are most relevant. The taxonomy is described as follows:

- Environmental uncertainty, which is associated with the environment that the observer cannot easily affect, mostly with the way the network and its sensors operate. The break-down provided below takes into account that the network (as the measurement instrument) is close to the reality of the physical world, but it is also a technological artefact that has a significant input from people.
- Aleatory uncertainty relates to the physicality of the situation, responding to the question “how exact can the measurement be”. This uncertainty is associated with the common experience of uncertainty in every measurement that contains some irreducible randomness, but also with uncertainty that results from the vagaries of the physical environment, such as temperature or humidity. This form of uncertainty is closely related to sensors as well as the physical phenomenon itself and to the environment in general. It is worth mentioning here that while the probabilistic approach is dominant in this area, care must be taken as harsh environmental conditions may easily invalidate this approach.
- Completeness uncertainty relates to the technological aspect of the network, i.e., to the fact that the network is an intentionally constructed artefact. This uncertainty attempts to answer the question “is this the best that could have been gathered”. It is associated with the understanding that selectiveness and imperfection are present throughout the network so that not every state of the phenomenon can be measured, collected, correctly transferred, stored or processed. Situations such as data loss, data corruption, duplication, temporal inversion, delays, etc. affect the completeness of information. Thus, there is an uncertainty whether the network captured what was important in a way that allows for further processing. This form of uncertainty is primarily related to data storage and transfer.
- Logical uncertainty relates to the social aspect of the network, i.e., to the fact, that the network is the expression of certain human logic (or possibly even several logics), together with their fallacies. Thus, situations such as conflicts, noise handling, cleansing, interpolation, etc. make the reconstruction contingent not only on data, but also on the logic derived from the expectation regarding the phenomenon. In addition, the introduction of novel analytical methods, e.g., the machine learning, created new challenges to uncertainty [49]. The question that may be asked here is “am I getting what was there, or what I expected to get”. This form is primarily related to the process of reconstruction, but it is also present, e.g., in relation to the embedded software that directly interacts with sensors.
- Systemic uncertainty is associated with the observer and relates to the fact that the observer does not observe objectively or in isolation, but always through socially developed constructs supported by the observer’s system. Here, further classification is guided by the understanding of the behavior of such a system [48]. It breaks down into three perspectives: towards itself, towards others and towards the future.
- Epistemic uncertainty relates to the system reflecting on itself, asking itself a question “do I know what I do”. It is associated with knowledge that is internalized by the system, whether codified as science or otherwise actively maintained within the system. This knowledge defines what is measured, how it is measured and how results are interpreted, yet it depends only on the social consensus that is neither perfect, not permanent. Thus, the system should accept the current state of its knowledge with uncertainty. Epistemic uncertainty affects all components of the model.
- Ethical uncertainty defines the view of the system towards specific others, whether they are people, other systems or technical devices. The ethics of this perspective relate to the key decision that the observer should make: whether the observer should trust the specific other or not. Such a decision is fraught with uncertainty, specifically when it comes to people, yet it has to be made to eliminate those components of the network that are not trusted.
- Utilitarian uncertainty relates to the system looking at its own future. As the system is aware that its existence may depend on getting correct information from the network, it remains in a state of uncertainty regarding whether what it receives is what it needs. This uncertainty comes from the anticipation that there is more to the phenomenon than theories covered, and there is knowledge that either is missing or whose existence is not even anticipated. Therefore, there may be a doubt whether results provided by the sensor network as a whole actually satisfy the observer.

One may notice that every category, under close scrutiny, may be recursively treated by the same or a very similar taxonomy to reveal even more forms and sources of uncertainty. For example, the uncertainty that relates to the logic applied to the reconstruction can be seen as being affected by the uncertainty related to the physical phenomenon, mostly the uncertainty of the distinction between the noise and the signal, the uncertainty of handling incompleteness of data, ethical uncertainty regarding intentions of implementers, etc. However, even the first level of such a taxonomy should be beneficial for structuring the discussion about IoT sensor networks.

### 3.3. Challenges in Uncertainty for IoT Networks

The model presented in Figure 1 can also serve to determine key challenges relevant to its various components. Those challenges are already indicated in Figure 1, next to relevant types of uncertainty. The challenges selected for the inclusion in this paper express the view of the authors and are here mostly to facilitate the discussion about uncertainty in IoT sensor networks.

It may be interesting that those challenges are mostly about developing better understanding of the construct of uncertainty through scientific as well as through practical means. It is also true that there are formidable challenges in developing better sensors or more reliable networks, but the authors believe that the success of IoT sensor networks is contingent less on the sheer quality of sensors and more on our ability to better assess, contain and relate to the uncertainty that IoT networks bring.

#### 3.3.1. Aleatory—Assessment

Statistical theories of uncertainty are established well enough to support the assessment of aleatory uncertainty. However, the new breed of sensors that are used in IoT measurement networks may require a different approach. Those sensors may not benefit from extensive calibration processes and may need to operate under the same rigorous protocols as their counterparts in professional networks. Thus, some of the assumptions of statistical approach may not be fully satisfied.

The benefits of taking those new devices under the umbrella of the unified statistical approach have already been seen in standardization activities. This is particularly true in the case of air quality sensors for the ambient environment, where the assessment of their uncertainty is governed by the data quality objectives (DQO) set in [50,51]. These objectives address the quality of the measurements by defining stringent protocols for the applied sensing methods (i.e., sampling, analysis and calibration), and they also introduce criteria concerning the highest relative expanded uncertainty accepted for the measurements. 

The uncertainty of the measurement is the most important set of criteria (DQO) which should be met in order for the measurements to be considered as equivalent to those that would have been achieved by using the so-called reference air quality (AQ) monitoring methods. Moreover, the uncertainty itself is used as a criterion that categorizes measurements into three “classes”: mandatory measurements (established via properly applied reference methods), indicative measurements and objective estimations, the latter corresponding to the highest uncertainty [52]. On this basis, important standardization work is underway, that is expected to lead to the first ever categorization of relevant IoT and stand-alone devices into categories in accordance with their measurement uncertainty.

#### 3.3.2. Completeness—Design

Sensor IoT networks do not benefit from the same quality of design process that is applied to professional networks. This may affect the completeness of data, increasing uncertainty associated with it. At the same time, the proliferation of low-cost sensors may add more data, with no guarantee that such overprovisioning will decrease such uncertainty. For this reason, recommendations say that relevant environmental quality measurements, like those assessing ambient air pollution, should reflect the overall size of the area of interest, the overall population as well as the population density, therefore introducing network design criteria that should be met. Moreover, areas of interest are divided into urban, suburban and rural, therefore introducing a land use criterion in the design of a relevant network. Next, for reasons of regulatory as well as scientific research oriented AQ monitoring, relevant locations (points of interest) are usually characterized as representative (i.e., reflecting the general conditions that demonstrate the spatial and temporal characteristics of an agglomeration), background (i.e., reflecting environmental conditions away from main pollution sources) and hotspot (i.e., revealing conditions dictated by local sources and human activity).

#### 3.3.3. Logic—Separation

Reconstruction faces a challenge of making a distinction between the value and the uncertainty associated with it. This distinction can be trivial for repeatable, well-defined measurements, where, e.g., the statistical approach will determine that the estimate of the mean value represents the actual value while the standard deviation represents uncertainty. The situation becomes more convoluted when the distinction between the value and its uncertainty becomes blurred, e.g., because little is known about the nature of such uncertainty. The approach taken in such situations is to assume certain characteristics of values and uncertainty, and to separate them according to such assumptions [5]. This approach is warranted, but only if such characteristics are well enough known.

IoT measurement networks find themselves in a peculiar position. Their growing complexity makes them gradually approach the point where the simple distinction may not be sufficient, yet the variety of sensors and solutions prevents any common characteristics from being established. Therefore, the challenge is about constructing the metric of uncertainty as well as the way of distinguishing uncertainty from values that do not rely on assumed characteristics but on the behavior of the network.

#### 3.3.4. Epistemic—Unification

It would be beneficial if there is one generally accepted metric and theory of uncertainty in IoT measurement networks that covers all components of such networks. Considering the current body of knowledge about uncertainty, specifically semantic differences between theories, such unification can be quite challenging and will require significant research effort.

It should be noted that it is not necessary for various theories to dispose of their views on uncertainty and the associated formal apparatus. The objective of the unification should not be conceptual unification, but rather the enablement of conversion between different theories that will lead to the single metric of uncertainty for the whole network.

It should be noted that such unification may also call for a more sociotechnical approach to uncertainty, possibly even incorporating the subjective side of it. Not indicating any particular direction, it is worth noticing that entropy (and the associated information-theoretic approach) may become a good candidate for such unification. Entropy already has strong links with the statistical approach as well as with other theories of uncertainty. However, other theories may claim similar development.

#### 3.3.5. Ethics—Trustworthiness

Ethical uncertainty deals with the doubt in intentions and in trustworthiness of various elements of the network, be it sensors, nodes or people. Currently, this area is mostly served through security-related research, where the focus is on identifying malicious nodes (see e.g., [44] for an overview of security problems relating to smart cities and IoT networks). It is not surprising because initial deployments of IoT networks, specifically low-power ones, suffered from security problems. 

However, generalizing trustworthiness as a computable quality of elements allows for extending this approach towards situations that are underserved by security alone, i.e., towards a more generalized view on uncertainty. For example, Ref. [35] uses trustworthiness to decrease uncertainty in some types of sensor networks. The same approach can be applied, e.g., to the “human aspect” of the network, i.e., to its operators, maintainers or designers, where both trust and uncertainty can be treated within the unifying framework (e.g., [53]).

#### 3.3.6. Utilitarian—Evolution

Measurement networks, whether professional or IoT, are constructed for a purpose, and they are useful only when they satisfy such purpose. The same applies to uncertainty, as it is the component of the relevance of the network. The authors of this paper believe that of the two, the greater challenge is relevance to keep the understanding of uncertainty in step with social needs, rather than perfecting it. As social needs change, so should the uncertainty.

The meaning of the construct of uncertainty is stabilized by social interactions, such as research, standardization, best practices, etc. Stabilization, however, does not imply petrification, but rather a gradual evolution of meaning in line with social understanding and needs. Theories of uncertainty, as well as the rest of the relevant body of knowledge, will become relevant to social needs through their change. The key challenge in this area is therefore the organization of the process of such changes. 

On this basis, one should take into account the standardization activities taking place at various levels, like the level of the physical sensor itself (the example of CEN/TC 264/WG 42 for Ambient air–Air quality sensors is of interest), the level of IoT sensor data access, management and characterization (SensorThings SWG of the Open Geospatial Consortium) and the level of information use under a Context Information Management umbrella (ETSI GR CIM 002 V1.1.1 (2018-09)). Such initiatives, although complementary, have not achieved a high level of consolidation yet, mainly because they originate from different parts of the industry and therefore have not been coordinated via a bottom-up approach.

## 4. Case Study

The primary objective of this paper is to facilitate the discussion of what progress should be made in regards to the theory and the practice of uncertainty of IoT sensor networks to satisfy current needs. However, even at this stage, this paper can be used to help structure actual problems and to suggest areas where development is desired.

For the sake of illustration, let us consider the example of the same citizen network that has been introduced early in this paper [1]. The model presented in this paper can help with the systematic identification of uncertainties that have to be dealt with, and with the most important research that should be done. The conceptual model of the sensor network (Figure 2), together with the associated taxonomy of uncertainty, allow identifying six key uncertainties within the network.

- Aleatory uncertainty, associated with the use of various sensors and measurement protocols, combined with the lack of proper maintenance and calibration.
- Collection uncertainty, associated with the opportunistic placement of sensors, with a certain disregard to the coverage.
- Logical uncertainty, associated with the use of algorithms of unknown impact on uncertainty.
- Epistemic uncertainty, which directly relates to the problem already expressed by the city hall: how to arrive at the single estimation of the uncertainty that will make the decision-making process feasible?
- Ethical uncertainty, that deals with the widely understood human factor, both within the network (i.e., the willing but inexperienced volunteer who sets wrong parameters), and outside of the network (i.e., the attacker who wants to alter the outcome of the measurements).
- Finally, there is the utilitarian uncertainty that deals with the question of whether what the network delivers actually helps solve the problem at hand.

The epistemic uncertainty, as indicated by the city hall, is the key one, and so is the challenge associated with it. It is, according to the paper, the need to unify different metrics of uncertainty. As a quick recollection, again guided by the model, in this IoT network, aleatory and collection uncertainties are likely to be dealt with using probabilistic approach, logical and epistemic uncertainties are managed using evidence theory, ethical approach may use Bayesian inference while the utilitarian one applies subjective reasoning.

As long as these uncertainties are incomparable, it is not possible to reason about or compute the uncertainty of the network. This paper and the model that it introduced indicate that there is a potential to consolidate various views on uncertainty using an information-theoretic approach and entropy. If this approach is not feasible, an alternative one aims at minimizing utilitarian uncertainty instead. This approach may, e.g., take on the form of improved standardization.

In summary, the model allowed for (1) systematic discovery of uncertainties; (2) structuration of the discussion about those uncertainties; (3) demonstration of their incomparability; (4) identification of the key challenge; and (5) indication of the direction in research that can help overcome this challenge.

## 5. Conclusions

The emergence of IoT measurement networks has brought about new challenges to our understanding of uncertainty. Their less stringent protocols, combined with lower quality sensors, ad hoc design and voluntary curation make some views and forms of uncertainty more important than they were in the case for professional networks. In order to progress the understanding of uncertainty in IoT sensor networks, it is necessary to develop new theories and practices across several disparate views on uncertainty. The development of a unified view on uncertainty in IoT networks is also desired.

This discussion paper has been structured to facilitate the development of various views on uncertainty, despite their differences. It provides a sociotechnical definition of uncertainty, describes a reference model of the IoT sensor network, maps the taxonomy of uncertainties to this model and defines challenges to uncertainty that the IoT measurement network creates. The case study, albeit brief, demonstrates how the model can be used to identify and structure problems regarding uncertainty in the actual IoT sensor network, and indicates directions that may deliver certain resolution.

IoT measurement networks are still in their infancy, leading to the focus on solving technical problems as well as on understanding their role in the decision-making process and the requirements it brings. Inevitably, the discussion will eventually move towards single assessment of the uncertainty of such networks, as it simplifies the comprehension of the value added by measurement networks.

The authors are currently working, among other topics, on the issue of the unification of various theories of uncertainty for the purpose of sensor networks, specifically IoT networks.

## Data Availability

Data sharing not applicable.
